# Immunophenotyping and activation status of maternal peripheral blood leukocytes during pregnancy and labour, both term and preterm

**DOI:** 10.1111/jcmm.13160

**Published:** 2017-04-21

**Authors:** Jianhong Zhang, Oksana Shynlova, Sally Sabra, Annie Bang, Laurent Briollais, Stephen J. Lye

**Affiliations:** ^1^ Lunenfeld‐Tanenbaum Research Institute Mount Sinai Hospital Toronto ON Canada; ^2^ Department of Obstetrics & Gynecology University of Toronto Toronto ON Canada; ^3^ Department of Physiology University of Toronto Toronto ON Canada

**Keywords:** pregnancy, peripheral leukocytes, term labour, preterm labour, immune activation

## Abstract

The onset of labour in rodents and in humans is associated with physiological inflammation which is manifested by infiltration of activated maternal peripheral leukocytes (mPLs) into uterine tissues. Here, we used flow cytometry to immunophenotype mPLs throughout gestation and labour, both term and preterm. Peripheral blood was collected from non‐pregnant women and pregnant women in the 1st, 2nd and 3rd trimesters. Samples were also collected from women in active labour at term (TL) or preterm (PTL) and compared with women term not‐in‐labour (TNIL) and preterm not‐in‐labour (PTNIL). Different leukocyte populations were identified by surface markers such as CD45, CD14, CD15, CD3, CD4, CD8, CD19 and CD56. Their activation status was measured by the expression levels of CD11b, CD44, CD55, CD181 and CD192 proteins. Of all circulating CD45+ leukocytes, we detected significant increases in CD15+ granulocytes (*i*) in pregnant women *versus* non‐pregnant; (*ii*) in TL women *versus* TNIL and *versus* pregnant women in the 1st/2nd/3rd trimester; (*iii*) in PTL women *versus* PTNIL. TL was characterized by (*iv*) increased expressions of CD11b, CD55 and CD192 on granulocytes; (*v*) increased mean fluorescent intensity (MFI) of CD55 and CD192 on monocytes; (*vi*) increased CD44 MFI on CD3+ lymphocytes as compared to late gestation. In summary, we have identified sub‐populations of mPLs that are specifically activated in association with gestation (granulocytes) or with the onset of labour (granulocytes, monocytes and lymphocytes). Additionally, beta regression analysis created a set of reference values to rank this association between immune markers of pregnancy and to identify activation status with potential prognostic and diagnostic capability.

## Introduction

The maternal immune system undergoes major transformation during pregnancy, likely impacted by the constantly changing hormonal environment. These changes are critical to maintain pregnancy and prevent the rejection of the foetal semi‐allograft 1, 2. Parturition is considered a localized physiologic inflammatory process 3. At the end of gestation, even in the absence of infection, the density of leukocytes in the uterine tissues (cervix, myometrium and decidua) increases, reaching the highest level at around term labour (TL) 4. Accumulating leukocytes promote cervical ripening (dilatation and effacement), take part in membrane/decidual activation and contribute to myometrial contractile activity, leading to expulsion of the baby and placenta 2. These events are preceded by an increase in the expression of uterine‐produced pro‐inflammatory cytokines and chemokines capable of activating maternal peripheral leukocytes (mPLs) 5 and inducing their infiltration into uterine tissues. Chemokine receptors are constitutively expressed on mPLs 6, 7, 8. It has been suggested that premature activation of the maternal immune system (*i.e*. cytokine secretion causing leukocyte influx) either by infection or by other risk factors can trigger premature cervical ripening, myometrial and/or decidual activation and preterm labour (PTL) leading to the delivery of a preterm baby 6, 9, 10. This assumption is supported by numerous findings: during PTL complicated by uterine infection, the human myometrium is infiltrated by immune cells 12; neutrophil abundance in the human decidua is elevated in PTL with infection as compared to corresponding controls; decidual macrophage abundance is higher in idiopathic PTL than in term, not‐in‐labour (TNIL) samples; and T cells and NK cells are more abundant in idiopathic PTL than TL 13. Furthermore, abnormal premature ripening associated with infection and inflammatory events usually results in PTL 14. PTL is an increasing public health problem and the leading cause of infant mortality in the world 15. It is associated with significant short‐term infant morbidity (respiratory distress syndrome, intraventricular haemorrhage, necrotizing enterocolitis, sepsis, bronchopulmonary dysplasia, retinopathy of prematurity, periventricular leukomalacia and patent ductus arteriosus) and long‐term illness (cerebral palsy, cognitive and behavioural deficits) 16, 17.

One of the first recognized gestation‐related changes in the peripheral blood is an increase in white blood cell (WBC) count 18, in particular, neutrophilia 19, 20, 21, 22, 23. Importantly, local changes in the uterus (term and preterm cervical ripening) may be reflected in the peripheral blood as an increase in WBC count 24. Several groups have reported that circulating leukocytes are activated in pregnant women without clinical signs of infection 25, 26, 27; for instance, WBC and C‐reactive protein were found to be significantly up‐regulated in labouring women compared to pregnant women not‐in‐labour 24. Changes in maternal peripheral blood indicate that normal pregnancy is associated with a continuous systemic inflammatory response 26. Indeed, we now know that the maternal inflammation is necessary for both the establishment and the completion of pregnancy. Increased WBC counts alone have been used to predict PTL (maternal WBC >12.0 mln/l at 22–27 weeks gestation with premature rupture of membranes (PROM) is the most accurate predictor of delivery within 48 hrs) 28. Furthermore, PTL with intact membranes is associated with phenotypic and metabolic changes in maternal granulocytes and monocytes 25, which display increased cytokine mRNA production and migratory ability 27. Thus, the activation status of mPLs likely plays a central role in determining pregnancy outcomes. Various proteins may be used to detect the activation status of mPLs. CD11b (also known as ITGAM) is a classic leukocyte activation marker, which mediates binding of mPLs to the vascular endothelium 29. Hyaluronic acid receptor CD44 has been shown to be involved in the recruitment of granulocytes and monocytes to the site of inflammation 30, 31. CD55 [decay‐accelerating factor (DAF)] is a complement inhibitory protein and multifunctional cell surface receptor presented on mPLs 32 and is elevated at the mRNA level in PLs during PTL 33.

Therefore, we hypothesize that an integrated analysis of the composition and activation status of mPLs during TL and/or PTL will provide informative data useful for screening and diagnosis of future PTL in asymptomatic women. Using flow cytometry (FC), we evaluated the expression and frequency of surface proteins CD11b, CD55, CD44, as well as chemokine receptors CD181 and CD192 on mPLs from non‐pregnant, healthy pregnant women throughout gestation (1st/2nd/3rd trimester), and patients admitted in labour (term or preterm). Regression analysis was used to rank the association between immune markers of mPLs and pregnancy, and to identify a sub‐set of activation markers with prognostic and diagnostic ability in determining a likelihood of premature delivery in high‐risk pregnant women.

## Materials and methods

### Study design

This prospective study was designed to compare the phenotypic characteristics of mPLs obtained from healthy pregnant women in the first trimester (gestational age (GA): 7–12 weeks), second trimester (GA: 13–26 weeks), third trimester (GA: 27–41 weeks) of gestation and term labour (GA: 39.3 weeks). Healthy females with regular physiological cycles were recruited as the non‐pregnant (NP) control group (Fig. S1A). In addition, we recruited women in labour at either term (GA≥37 weeks; TL) or preterm (GA: 24–37 weeks; PTL); healthy pregnant women matched for GA were recruited as term controls (≥37 weeks; TNIL) and preterm controls (24–37 weeks; preterm not‐in‐labour, PTNIL) (Fig. S1B). This study was approved by the Research Ethics Board at Mount Sinai Hospital (MSH), Toronto, Canada. Informed consent was obtained from the patients before their participation in the study. All pregnant women participating in this study were recruited from MSH inpatient clinics and labouring patients were recruited upon admission to the Department of Obstetrics and Gynecology, High Risk Antenatal Clinic and/or Labor and Delivery ward, MSH, Toronto. Clinical data are presented in Table 1.

**Table 1:** (A) Patients’ demographics of Study 1. (B) Patients‘ demographics of Study 2

(A)	Non‐pregnant	1st Trimester	2nd trimester	3rd trimester	Term labour
Number of samples	10	10	29	40	38
Maternal Age (mean ± SD; year)	28.9 ± 8.1	27.2 ± 7.6	33.7 ± 4.2	35.0 ± 4.6	33.7 ± 5.2
Gravidity (Median, range)	0 (0–2)	1 (1–2)	1 (1–4)	2 (1–7)	2 (1–7)
Parity (Median, range)	0 (0–2)	0 (0–2)	0 (0–2)	1 (0–4)	0 (0–3)
Gestational Age (mean ± SD; week)	N/A	9.3 ± 1.5	20.0 ± 2.2§	36.9 ± 3.8*	39.3 ± 0.9*

(B)	PTNIL	PTL	TNIL	TL
Number of samples	15	18	35	38
Maternal Age (mean ± SD; year)	33.5 ± 5.2	36.4 ± 5.7	35.4 ± 3.9	33.7 ± 5.2
Gravidity (Median, range)	1 (1–4)	2 (1–7)	2 (1–7)	2 (1–7)
Parity (Median, range)	0 (0–1)	0 (0–2)	1 (1–4)	0 (0–3)
Gestational Age (mean ± SD, week)	27.9 ± 2.6	29.7 ± 4.1	38.9 ± 0.9†	39.3 ± 0.9†

§Indicates statistical difference between 1st and 2nd trimester, *P* < 0.05.

*Indicates statistical difference between 2nd and 3rd trimester, 2nd trimester and term labour (TL) *P* < 0.05.

†Indicates statistical difference between preterm not‐in‐labour (PTNIL) *versus* term not‐in‐labour (TNIL) and preterm labour (PTL) *versus* TL, *P* < 0.05.

### Inclusion criteria

Participants were 16–45 years old healthy women with singleton pregnancy (Table 1). The diagnosis of labour was made when the patients had regular uterine contractions associated with cervical changes and cervical dilation more than 4 cm. Spontaneous PTL with intact membranes was diagnosed in women with at least eight uterine contractions in one hour and cervical changes at a gestational age of <37 weeks. All PTL patients delivered in the following 48 hrs. The NP control group consisted of healthy women of similar age.

### Exclusion criteria

Patients with PPROM, IVF pregnancies, inflammation and/or infection (chorioamnionitis), multiple pregnancy, foetal anomalies, gestational diabetes, cervical cerclage, gestational hypertension, preeclampsia, antepartum haemorrhage and history of any autoimmune disorder were excluded from the study.

### Study populations

*Study 1* was used to assess gestational changes in the activation status of PLs (Table 1A). Healthy women with singleton pregnancies with no medical or obstetrical complications were enrolled throughout gestation in the 1st trimester (*n* = 10), 2nd trimester (*n* = 29), 3rd trimester (*n* = 40) of gestation and labour (*n* = 38). Healthy non‐pregnant females are included as a control group (*n* = 10).

*Study 2* was used to assess specific labour‐related changes (Table 1B). Healthy term women with singleton pregnancies and no sign of labour (TNIL, *n* = 35) were compared to healthy patients in active labour (TL, *n* = 38); patients experiencing idiopathic PTL (*n* = 18) were compared with gestational age‐matched healthy women not‐in‐labour PTNIL (*n* = 15).

### Blood samples

Peripheral blood was collected from patients upon admission to the hospital or during the regular visit to the antenatal clinic. Samples were collected by venipuncture into 5 ml Cyto‐Chex^®^ blood collection tubes that contain a patent‐protected preservative maintaining the integrity of cellular CD markers for immunophenotyping by FC for up to seven days. The FC analysis was performed within 2–72 hrs following blood collection.

### Flow cytometry staining protocol

Direct immunofluorescence staining of peripheral blood using a lyse/no‐wash procedure was applied. In brief, the whole blood (25 μl) was mixed with an equal volume of blocking solution (Serum Free Protein Block, Dako, CA, USA) and incubated for 20 min. at room temperature (RT). The specific antibodies (Table 2) were added at the concentration determined by preliminary experiments, and incubated for 30 min. at RT in the dark. Freshly prepared FACS Lyse solution (450 μl; BD Bioscience, San Jose, CA, USA) was added to each tube and incubated for 15 min. prior to data acquisition by FACSAria (BD Biosciences) or by Gallios flow cytometer (Beckman Coulter, Brea, CA, USA).

**Table 2:** Monoclonal mouse anti‐human antibodies used for the flow cytometry

Antibodies	Fluorochrome	Manufacturer	Catalogue number	Leukocyte specificity
CD45	APC‐H7	BD Pharmingen	641399	Leukocytes
CD14	PerCP	BD Pharmingen	340585	Monocytes
CD15	FITC	BD Pharmingen	555401	Granulocytes
CD3	FITC	BD Pharmingen	555339	T lymphocytes
CD4	APC	BD Pharmingen	555349	Helper T cells
CD8	PerCP	BD Pharmingen	347314	Cytotoxic T cells
CD19	APC	BD Pharmingen	555415	B lymphocytes
CD16	PE	BD Pharmingen	347617	NK cells
CD56	PE‐Cy7	BD Pharmingen	557747	NK cells
CD11b	PE‐Cy7	BD Pharmingen	557743	β2 integrin (Mac‐1)
CD44	PE	BD Pharmingen	550989	Cell surface HA
CD55	PE	BD Pharmingen	555694	Complement activation marker
CD181	PE	BD Pharmingen	555940	IL‐8 receptor
CD192	Alexa Fluor647	BD Pharmingen	558406	MCP‐1 receptor

### Flow cytometric data analysis

FlowJo V10 (TreeStar, Inc., Ashland, OR, USA) or Kaluza 1.3 (Beckman Coulter) software was used for the offline data analysis. Leukocytes were identified by their characteristic forward and side light scatter properties. CD45 trigger threshold was applied. Gating strategy used to identify different sub‐populations of leukocytes (CD45+CD15+ granulocytes, CD45+CD14+ monocytes and CD45+ CD3/19/56+ lymphocytes) is depicted in Figure S2. The frequency and the mean fluorescence intensity (MFI) of CD11b, CD44, CD55, CD181 and CD192 expression by different leukocyte sub‐populations were assessed (Fig. S3).

### Statistical analysis

Statistical analysis was performed by SPSS23 (IBM, Armonk, NY, USA) and R software (version 3.2.3) using Betareg package 34. To determine pregnancy‐related leukocyte activation status, multiple comparisons were conducted between study groups by Kruskal–Wallis test followed by Mann–Whitney *U* test. Beta regression of leukocyte frequency is calculated by R package (betareg) to correlate leukocyte activation status with clinical factors. Multiple regressions were performed using SPSS to evaluate the association between MFI of leukocyte activation markers and GA/labour. Statistical significance was assumed when *P* < 0.05.

## Results

### Maternal characteristics

Clinical characteristics of non‐pregnant, pregnant and labouring patients are shown in Table 1. For the healthy pregnant women (1st/2nd/3rd trimester), there was no difference in age, parity and gravidity; only gestational age was significantly different between groups (*P* < 0.05, Table 1A). Patients in spontaneous PTL with intact membranes and those in the control PTNIL group were matched for gestational age; similarly, TL patients were matched for gestational age with TNIL women (Table 1B). Gestational age of patients in preterm groups was different from that of the term groups (*P* < 0.05). All patients in the TNIL group underwent elective or repeat C‐Section delivery prior to labour onset.

### Effect of gestation on maternal peripheral leukocytes

**Figure 1:**
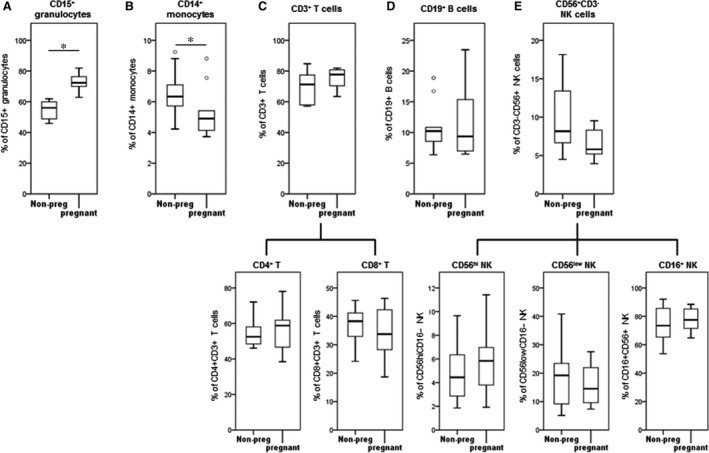
Percentage of CD45+ leukocyte sub‐sets in peripheral blood from non‐pregnant and pregnant women. Peripheral blood was collected into Cyto‐Chex tubes and stained with different leukocyte markers (CD14, CD15, CD3, CD4, CD8, CD19, CD56 and CD16) to identify (**A**) granulocytes, (**B**) monocytes, (**C**) T cells, (**D**) B cells and (**E**) NK cells. Flow cytometry data were acquired by Gallios cytometer followed with analysis by Kaluza 1.3 software. The distribution of different leukocyte sub‐sets (% from total CD45+ leukocytes) was calculated. Data are presented as mean ± SD. Significant difference between non‐pregnant and pregnant women is indicated by *, *P* < 0.05.

**Figure 2:**
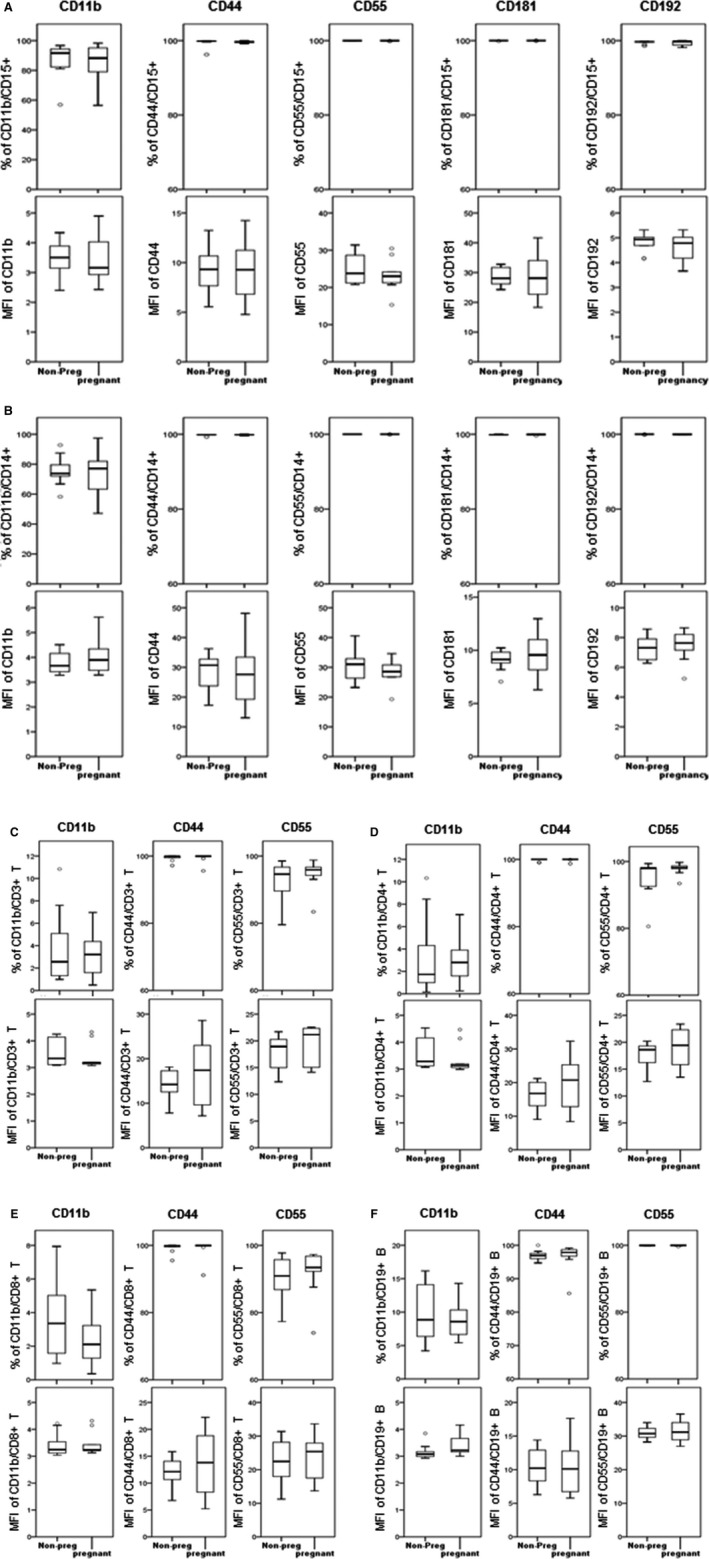
The activation status of circulating CD45+ leukocytes in peripheral blood of non‐pregnant and pregnant women. Peripheral blood was collected in Cyto‐Chex tubes and stained with leukocyte surface and activation markers (CD11b, CD44, CD55, CD181 and CD192) to assess the immunological characteristics of (**A**) CD15+ granulocytes (**B**) CD14+ monocytes, (**C**) CD3+ T cells, (**D**) CD4 + CD3+ T cells, (**E**) CD8+CD3+ T and (**F**) CD19+ B cells. The percentages of positive sub‐sets (top panel) and their signal intensity mean fluorescent intensity (MFI) (bottom panel) are shown as box plots. Flow cytometry data were acquired by Gallios cytometer followed with analysis by Kaluza 1.3 software. Significant difference between non‐pregnant and pregnant women is indicated, when applicable, by *, *P* < 0.05.

The isolation of leukocytes from peripheral blood may activate them and change immune features not naturally presented *in vivo* 26. Therefore, we collected maternal blood samples in the CytoCheck BCT specifically designed to protect leukocyte surface proteins. Using carefully selected patient populations and ‘whole blood/no lyse’ protocol for FC analysis, we applied CD45/SSC gating to ensure accuracy and identify novel activation markers on mPLs (Fig. S2). Of all circulating CD45+ leukocytes, we detected a significant increase in CD15+ granulocytes and a significant decrease in CD14+ monocytes in pregnant (1st trimester) as compared to non‐pregnant women (Fig. 1A and B). There was no significant difference detected in lymphocyte sub‐populations between non‐pregnant and pregnant women (Fig. 1C–E). The expression of activation markers (CD11b, CD44, CD55, CD181 and CD192) on the leukocyte sub‐sets was similar between the two groups (Fig. 2).

### Maternal peripheral leukocytes activation during pregnancy

**Figure 3:**
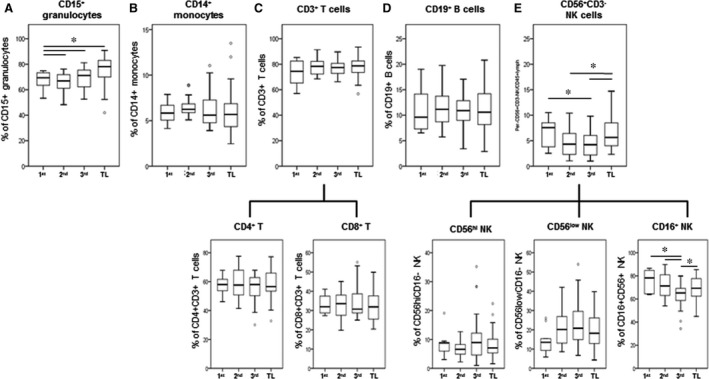
The change in percentage of circulating CD45+ leukocytes in peripheral blood of pregnant women throughout gestation (1st/2nd/3rd trimester and post‐dates) and term labour (TL). Peripheral blood were collected in Cyto‐Chex tubes and stained with different leukocyte markers (CD14, CD15, CD3, CD4, CD8, CD19, CD56 and CD16) to identify (**A**) granulocytes, (**B**) monocytes, (**C**) T cells, (**D**) B cells and (**E**) NK cells. Flow cytometry data were acquired by FACSAria cytometer followed with analysis by Flowjo V10 software. The distribution of different leukocyte sub‐sets (% from total CD45+ leukocytes) was calculated. Data are presented as mean ± SD. Significant difference between early and late gestation as well as between pregnant and labouring women is indicated by *, *P* < 0.05.

**Figure 4:**
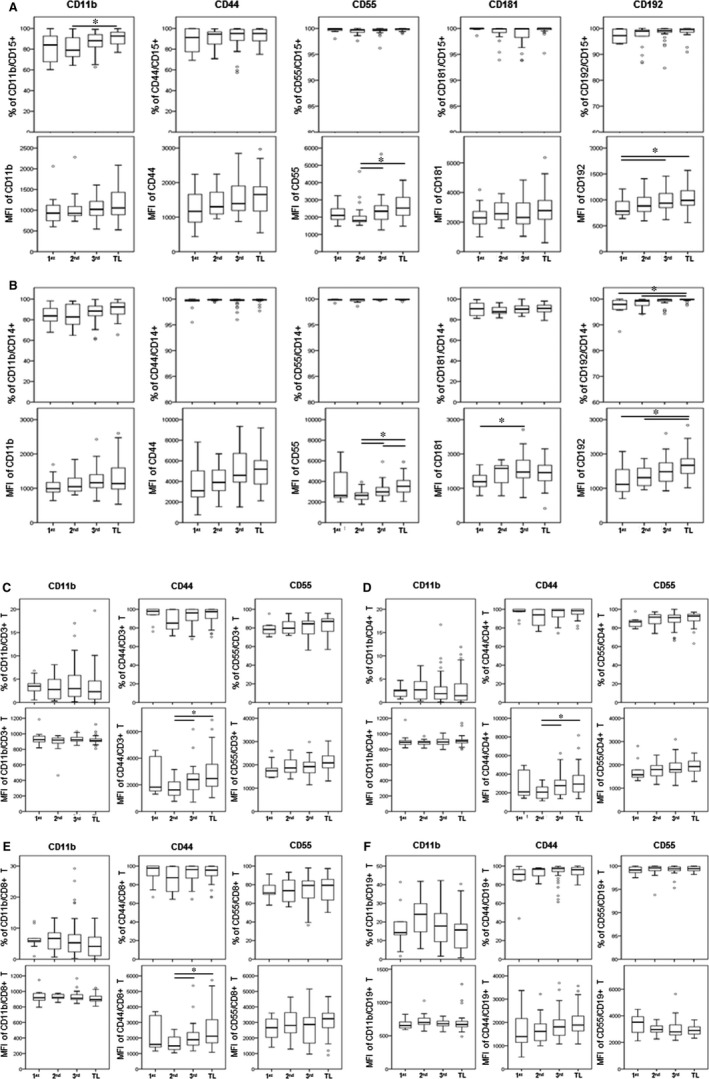
The activation status of circulating CD45+ leukocytes in maternal blood of pregnant (1st/2nd/3rd trimester and post‐dates) and term labouring (TL) women. Peripheral blood was collected in Cyto‐Chex tubes and stained with leukocyte surface markers (CD14, CD15, CD3, CD4, CD8 and CD19) and activation markers (CD11b, CD44, CD55, CD181 and CD192) to assess activation status of (**A**) CD15+ granulocytes, (**B**) CD14+ monocytes, (**C**) CD3+ T cells, (**D**) CD4+CD3+ T cells, (**E**) CD8+CD3+ T cells and (**F**) CD19+ B cells. The percentages of positive sub‐sets (top panel) and their signal intensity mean fluorescent intensity (MFI) (bottom panel) are shown as box plots. Flow cytometry data were acquired by Gallios cytometer and are presented as mean ± SD. Significant difference in the activation markers between early and late gestation as well as between pregnant and labouring women is indicated by *, *P* < 0.05.

Analysis of the frequencies of mPL sub‐sets indicates a significant increase in percentage of circulating granulocytes across gestation and during TL *versus* 1st trimester of pregnancy (*P* < 0.05, Fig. 3). We observed a significant increase in the proportion of CD15+ granulocytes expressing CD11b (*P* < 0.05) during TL compared with granulocytes from 2nd trimester women (Fig. 4A). The majority of granulocytes were positive for cell surface markers CD44, CD55, CD181 and CD192 and there were no significant differences in the number of expressing cells between 1st/2nd/3rd trimester and TL. However, we detected a significant increase in the MFI levels of CD192 and CD55 on CD15+ granulocytes during TL or 3rd trimester as compared to 1st or 2nd trimester, respectively (Fig. 4A), which indicates increased detection of these two proteins on the surface of granulocytes and increased sensitivity to CCL2 cytokine activation (CD192) and complement activation (CD55).

In contrast to previous reports 20, 27, we did not detect any differences in the percentage of monocytes in TL blood samples compared to 1st/2nd/3rd trimester samples (Fig. 3B). Almost all monocytes expressed each of the cell surface markers studied. There was no difference in the percentage of monocytes expressing CD11b, CD44, CD55 and CD181; however, the number of CD192+ monocytes was significantly up‐regulated during TL as compared to 1st/2nd trimester of pregnancy (Fig. 4B) which could promote monocyte infiltration into the uterine tissues in response to labour‐stimulated CCL2 levels. Moreover, monocytes were activated before and during TL: MFI levels of CD55 were significantly elevated from 2nd to 3rd trimester, and they were the highest during TL (*P* < 0.05); similarly, the MFI of CD192 was significantly higher during TL as compared to 1st and 2nd trimester (*P* < 0.05), whereas MFI level of CD181 was elevated in 3rd trimester compared to 1st trimester (*P* < 0.05, Fig. 4B).

Our current experiments did not detect significant differences in the number of CD3+/CD4+/CD8+ T lymphocyte and CD19+ B lymphocytes between pregnant and TL women (Fig. 3C and D); however, peripheral CD56+CD3− NK cells (in particular, the CD16+ pNK sub‐population) show a significant decrease from 1st to 3rd trimester, with a slight (but significant) increase during TL (Figure 3E). The proportional changes of peripheral NK sub‐sets indicate that these cells are actively involved in the immunosuppression and tolerance responding to stage‐specific foetal development. There was no significant difference in the numbers of T and B lymphocytes expressing CD11b, CD44 and CD55 between 1st/2nd/3rd trimester and TL; only the MFI for CD44 of both CD3+/CD4+ and CD3+/CD8+ T lymphocytes was significantly up‐regulated starting from 2nd trimester until TL (Fig 4C–E).

### Effect of term and preterm labour on maternal peripheral leukocytes

**Figure 5:**
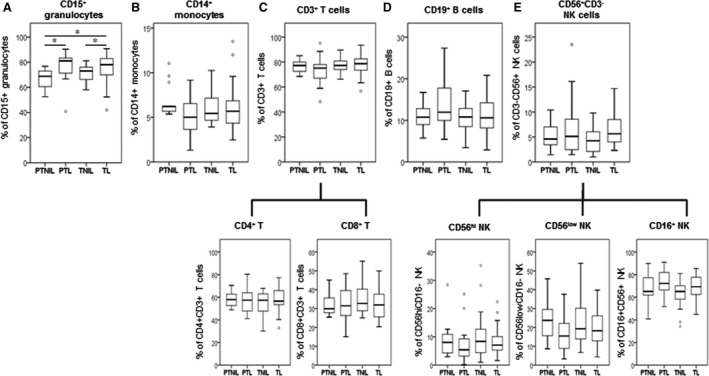
Changes in the proportion of circulating peripheral CD45+ leukocytes during human labour, preterm and term. Peripheral blood was collected in Cyto‐Chex tubes and stained with a variety of leukocyte markers (CD14, CD15, CD3, CD4, CD8, CD19, CD56 and CD16) to identify (**A**) granulocytes, (**B**) monocytes, (**C**) T cells, (**D**) B cells and (**E**) NK cells. Flow cytometry data were acquired by FACSAria cytometer followed with analysis by Flowjo V10 software. The percentage of each leukocyte sub‐population (% from total CD45+ leukocytes) was calculated. Data are presented as mean ± SD. Significant difference between labouring and non‐labouring women is indicated by *, *P* < 0.05.

**Figure 6:**
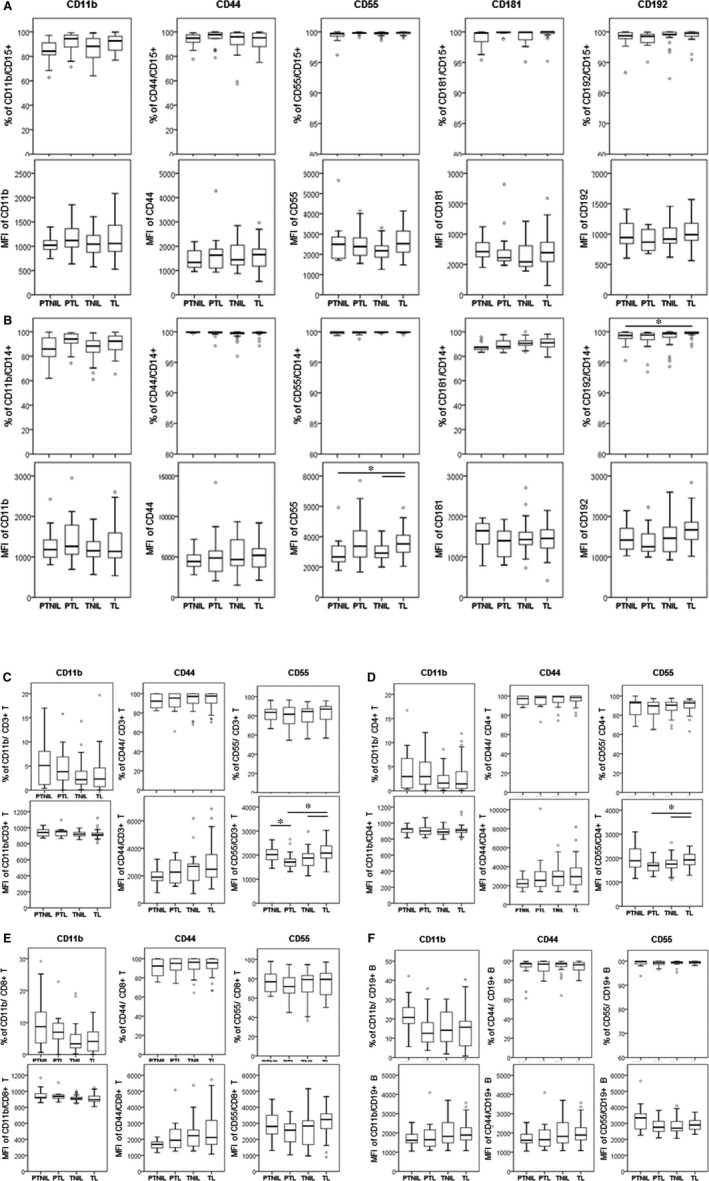
The activation status of circulating leukocytes among preterm not‐in‐labour (PTNIL), PTL (PTL), term not‐in‐labour (TNIL) and term labour (TL) pregnancy. Peripheral blood was collected in Cyto‐Chex tubes and stained with leukocyte surface markers (CD14, CD15, CD3, CD4, CD8 and CD19) and activation markers (CD11b, CD44, CD55, CD181 and CD192) to assess the activation status of (**A**) CD15+ granulocytes, (**B**) CD14+ monocytes, (**C**) CD3+ T cells, (**D**) CD4+CD3+ T cells, (**E**) CD8 + CD3+ T cells and (**F**) CD19+ B cells. The percentages of positive sub‐sets (top panel) and their signal intensity mean fluorescent intensity (MFI) (bottom panel) are shown as box plots. Flow cytometry data were acquired by Gallios cytometer followed with analysis. *, *P* < 0.05.

Granulocytes were increased in blood from labouring women both term and preterm. Patients admitted in active TL had significantly higher percentage of CD15+ granulocytes compared to gestational age‐matched patients from TNIL; idiopathic PTL patients had higher circulating levels of CD15+ granulocytes compared to PTNIL women (Fig. 5A, *P* < 0.05 for both). In contrast to previous reports 20, 27, no significant difference was detected in number of monocytes or lymphocytes either between TL and TNIL, or PTL and PTNIL women matched for gestational age (Fig. 5B–E). There was also no labour‐related change found in the number of mPLs expressing activation markers such as CD11b, CD44, CD55, CD181 and CD192, except for the significant increase in the number of CD192+ monocytes during TL compared to PTNIL, demonstrating their increased preparedness to infiltrate into CCL2‐producing uterine tissues. MFI values for CD11b, CD44, CD181 and CD192 were not changed by TL or PTL. The only significant increase related to labour status was recorded in MFI for CD55 of CD14+ monocytes and CD3+, CD4+ T lymphocytes, indicating their activation during TL relative to TNIL and PTNIL (*P* < 0.05, Figs 5 and 6).

### The association between pregnancy, labour and leukocytes activation

We used beta regression methods to model the association of different leukocyte sub‐types with gestational age or labour status (Table 3). Of all measured immunological parameters, the percentage of CD16 + CD56+ NK lymphocytes (the major component of pNK) is the only group of mPLs that has strong association to both clinical variables, that is, gestational age and labour status (*P* < 0.05). Gestational age contributed significantly to the proportion of circulating CD192+/CD44+/CD181+/CD14+ monocytes, CD11b+CD3+, CD11b+CD8+ T lymphocytes, CD19+ B lymphocytes and CD192+CD15+ granulocytes (*P* < 0.05). The onset of labour impacts the proportion of CD15+ granulocytes, CD14+ monocytes and CD56^low^ NK/CD56+CD3− NK cells (*P* < 0.05). As reflected by the pseudo‐*R* ^2^ statistics, the frequency of blood CD15+ granulocytes is most strongly associated with gestational age and labour status, indicating their increase with the progression of pregnancy and onset of labour. The frequency of CD44 and CD55 expression by CD4+/CD8+/CD3+ T cells, however, has limited the association with gestational age and labour status, as illustrated by the smaller pseudo‐*R* ^2^ value and larger predictive probability. NK sub‐sets, featured as CD56+CD3−, CD16+CD56+ and CD56^low^, have relatively higher values of pseudo‐*R* ^2^ compared to other leukocyte populations, indicating that peripheral NK lymphocytes (pNK) are sensitive to gestational changes.

**Table 3:** Beta regression of leukocytes frequency using R program

Variables	Pseudo‐*R* ^2^	*P*|_Gestation Age	*P*|_labour
% CD15+ granulocytes	0.131	0.834	0.000173***
% CD16+CD56+ NK	0.114	0.0314*	0.00524**
% CD192+/CD14	0.098	6.41e‐05***	0.988
% CD11b+/CD8	0.093	0.00243**	0.671
% CD56^low^ NK cells	0.066	0.252	0.0255*
% CD44+/CD14	0.064	0.0141*	0.598
% CD56+CD3− NK cells	0.063	0.586	0.0196 *
% CD14+ monocytes	0.059	0.401	0.0273*
% CD11b+/CD3	0.058	0.0270*	0.640
% CD55+/CD15	0.053	0.412	0.00624**
% CD11b+/CD19	0.049	0.115	0.087
% CD11b+/CD4	0.047	0.080	0.592
% CD19+ B cells	0.042	0.0493*	0.300
% CD56^hi^ NK cells	0.036	0.140	0.346
% CD181+/CD15	0.032	0.668	0.178
% CD192+/CD15	0.032	0.0357*	0.850
% CD55+/CD19	0.031	0.052	0.301
% CD44+/CD15	0.025	0.259	0.610
% CD11b+/CD14	0.023	0.347	0.340
% CD55+/CD14	0.020	0.209	0.561
% CD11b+/CD15	0.019	0.718	0.286
% CD8+ T cells	0.018	0.930	0.186
% CD181+/CD14	0.018	0.0371*	0.385
% CD44+/CD3	0.015	0.301	0.593
% CD4+ T cells	0.013	0.679	0.275
% CD3+ T cells	0.012	0.256	0.775
% CD44+/CD19	0.012	0.360	0.608
% CD44+/CD8	0.005	0.635	0.588
% CD55+/CD3	0.005	0.857	0.512
% CD55+/CD4	0.003	0.759	0.485
% CD44+/CD4	0.001	0.994	0.815
% CD55+ CD8	0.001	0.815	0.834

A beta regression was conducted to predict frequency of leukocytes from gestational age and labour status using R program (betareg). Pseudo *R*‐squared was listed to evaluate the goodness‐of‐fit of logistic models when logit link was applied. Significant codes of *P* (probability) are: 0, ‘***’; 0.001, ‘**’; 0.01, ‘*’.

In addition, a multiple regression was performed to evaluate the association between MFI levels of mPLs and gestational age or labour status (Table 4). MFI values of CD55 expression on CD14+ monocytes and CD11b expression on CD8+ T cells have higher pseudo‐*R* ^2^ value than other MFI measurements indicating that CD55 protein density on these cells is a critical parameter to be measured for the predictive study of pregnancy outcomes. Specifically, the signal intensity of CD55 on CD14+ monocytes is significantly associated with labour while gestational age has great contribution to MFI of CD11b+CD8+ T lymphocytes (*P* < 0.05). This suggests that relative signal intensity of CD55 and CD11b, rather than their frequency (*i.e*. ‘%’), should be used for the assessment of monocytes and T lymphocytes activation throughout gestation. Also, gestational age has a significant contribution to the MFI changes of CD11b expression on CD3+ T lymphocytes and CD192 expression on CD14+ monocytes (*P* < 0.05) indicating their increased capability to infiltrate into uterine tissues with approaching labour.

**Table 4:** Multiple regression analysis of MFI values using SPSS statistics

Variables	Pseudo‐*R* ^2^	Sig|_Gestation Age	Sig|_labour
MFI_CD55/CD14+	0.070	0.897	0.003*
MFI_CD11b/CD8+	0.067	0.005*	0.660
MFI_CD44/CD3+	0.044	0.052	0.147
MFI_CD192/CD14+	0.039	0.046*	0.316
MFI_CD44/CD8+	0.032	0.122	0.114
MFI_CD181/CD15+	0.028	0.099	0.133
MFI_CD11b/CD3+	0.026	0.037*	0.586
MFI_CD11b/CD14+	0.024	0.164	0.106
MFI_CD11b/CD15+	0.012	0.616	0.085
MFI_CD55/CD15+	0.010	0.557	0.102
MFI_CD55/CD19+	0.010	0.101	0.774
MFI_CD44/CD4+	0.000	0.606	0.190
MFI_CD192/CD15+	0.000	0.167	0.973
MFI_CD181/CD14+	−0.002	0.970	0.185
MFI_CD55/CD3+	−0.006	0.447	0.367
MFI_CD11b/CD4+	−0.008	0.711	0.289
MFI_CD44/CD19+	−0.014	0.456	0.758
MFI_CD55/CD4+	−0.017	0.565	0.704
MFI_CD55/CD8+	−0.017	0.671	0.579
MFI_CD44/CD15+	−0.018	0.764	0.657
MFI_CD11b/CD19+	−0.021	0.935	0.765
MFI_CD44/CD14+	−0.021	0.900	0.908

MFI, mean fluorescent intensity. A multiple regression was performed by SPSS23 to predict MFI levels of leukocytes from gestational age and labour status. The method of ‘ENTER’ was applied. Significant codes (Sig): 0.01, ‘*’.

## Discussion

Through a detailed immunophenotyping technique, we identified two new protein markers (CD44 and CD55) that could be used to study activation status of peripheral leukocytes in different clinical and experimental settings. Our data demonstrate that (1) normal pregnancy caused a significant increase in granulocyte frequency which peaked during active TL; (2) normal pregnancy was associated with activation of different leukocyte subtypes—granulocytes (by CD11b and CD55), monocytes (by CD55, CD181 and CD192) and T lymphocytes (by CD55 and CD44); (3) during spontaneous PTL, granulocyte numbers were significantly increased but their activation markers were not changed. We conclude that normal human gestation is manifested by physiologic activation process detectable in maternal blood, which increases during pregnancy and the progression to labour.

Granulocytes, specifically neutrophils, make up a large fraction of PLs representing the ‘first line of defence’ against infections 35. We report here a pregnancy increase in the proportion of peripheral granulocyte in maternal blood (compared to NP) implying a functional role in these reproductive processes. Granulocytes gradually and significantly increased from 1st to 3rd trimester in the peripheral blood of pregnant women culminating during TL 18, 20, 36. Our data support these findings indicating that during both TL and PTL, peripheral blood neutrophil numbers are increased and their homing into the uterine tissues is elevated 4, 12, 18, 22, 26. Multiple studies using morphological gating suggest that granulocytes may contribute to the process of labour 24, that premature and prolonged labour is associated with higher percentages of maternal granulocytes than TL 25, 26. In our study, the proportion of CD45+CD15+ granulocytes was up‐regulated by labour, which suggests they may contribute to labour induction, by producing inflammatory mediators, including plasminogen activators, eicosanoids, pro‐inflammatory cytokines (*i.e*. IL‐1β and TNF‐α) 37 and extracellular matrix degrading proteases such as neutrophil elastase, neutrophil collagenase (MMP‐8) and gelatinase MMP‐9 38.

Monocytes are versatile cells that play a central role in innate and adaptive immunity. They represent a small fraction of mPLs (5–6%) capable of migrating to specific anatomical locations, and upon exposure to the local tissue microenvironment, to differentiate into tissue‐specific macrophages 39. There is some controversy about the percentage of monocytes during pregnancy and labour between our current results and others 20, 27. However, there is no doubt that as a reservoir of inflammatory cytokines within mPLs, monocytes participate in a wide range of biological processes, including initiation and resolution of inflammation, stimulation of differentiation, and activation of other PL sub‐populations, that is, lymphocytes 40, 41.

The trafficking of mPLs through the body is controlled by their interactions with the vascular endothelium. To facilitate transmigration into target organs, circulating leukocytes must express integrin molecules macrophage‐1 antigen (Mac‐1, αMβ2, CD11b/CD18) and LFA‐1 on their surface 35, 42. CD11b mediates binding of leukocytes to intercellular adhesion molecule (ICAM‐1/CD54) that is up‐regulated in the uterine endothelium during human TL 29. CD11b has an extensive intracellular storage pool that could be released to the cell surface with activation or excessive manipulation 43. In consistency with previous studies 25, 27, 47, our current data similarly show that there is a proportional increase in the expression of CD11b on granulocytes during TL which indicates their increased ability to migrate towards chemotactic signal produced by uterine tissues. In line with other reports 20, 27, 44, we found that the majority of CD14+ monocytes are CD11b positive although no significant pregnancy‐ or labour‐related changes in CD11b MFI levels were found. The reason for this discrepancy may relate to different methods for blood collection and antibodies used for FC analysis, as the MFI measure is sensitive to biological, experimental and instrumental settings.

Our data provide the first evidence that surface expression of CD44 is up‐regulated throughout human gestation on granulocytes, monocytes, T and B lymphocytes; however, statistical significance between pregnant women and TL was only found for CD3+ T cells. In contrast to Yuan *et al*. 27, we did not record a greater proportion of CD4+ or CD8+ T cells in peripheral blood of PTL women when compared to PTNIL. However, we did detect a significant increase in the MFI of CD44 on the surface of CD3+ T lymphocytes during TL. Both T lymphocyte sub‐populations, cytotoxic T cells (CD8+) and helper T cells (CD4+), expressed higher CD44 throughout gestation and TL. Given the crucial role for leukocyte–endothelial and leukocyte–leukocyte interaction, we concluded that both, CD11b and CD44, may facilitate the recruitment of mPLs into uterine tissue during TL. In support of this assumption, we reported earlier that expression of both CD11b and CD44 was increased on granulocytes and monocytes after incubation with media, conditioned by mechanically stretched human myometrial cells that contained multiple inflammatory cytokines and chemokines (CXCL8, CXCL1, etc.) 44, 45. As the major cellular components of the adaptive immune response, up‐regulation of CD44+ T cells can potentially boost CD3+CD25+ regulatory T‐cell functions, which have a critical role in controlling maternal–foetal immune tolerance and inflammatory response 46, 47, 48.

It has become evident that membrane complement regulatory proteins are important in the maintenance of pregnancy. For instance, CD55^−/−^ mice die *in utero* from placental inflammation, demonstrating the critical protective role of CD55 50. Recent studies showed an elevated level of anaphylatoxin C5a in plasma of women with PTL (but not in TL), indicating that complement activation is different between term and preterm parturition 51. Moreover, CD55 mRNA expression was elevated on mPLs of PTL patients compared with gestational age‐matched PTNIL pregnant women 33. Thus, we analysed in detail CD55 protein expression on mPLs of pregnant and labouring patients and detected CD55 protein expression by all immune cell sub‐types, which highlights the importance of protecting self‐tissues from complement cascade activation. In our experiments, CD55 protein density was increased on monocytes and granulocytes during TL compared to 2nd/3rd trimester of gestation and on CD3+CD4+ lymphocytes during TL *versus* TNIL, indicating that CD55 actively protects leukocytes from complement‐mediated membrane damage.

Our present data also confirm previous publications indicating that T and B lymphocytes are not significantly changed, but we found the proportion of CD56+CD3− and CD16+ NK cells decreases significantly with pregnancy 52, 53. Furthermore, our regression analysis identified the relative importance (pseudo‐*R* ^2^ values) of immune activity of different leukocyte subtypes. We found that CD15+ granulocytes and CD16+CD56+ NK cells had relatively higher association with labour induction than CD3+/4+/8+T or CD19+ B cells. These statistical results are consistent with previous reports 55, 56, 57 suggesting that both granulocytes and NK cells are critical for the systemic immune regulation in human pregnancy and may function synchronously to sense the maternal or foetal changes associated with labour induction. We speculate that peripheral NK cells could be progressively primed during pregnancy, a feature identified during viral infection 57; furthermore, different NK sub‐sets (CD56+CD3−, CD56^hi^, CD56^low^ and CD16 + CD56+) may be selectively activated in response to gestation or labour. Such coordinated immune regulations could benefit the stabilization of maternal–foetal tolerance and prevent NK‐related pregnancy failure 58, 59. Unfortunately, current study did not include other surface NK activation markers previously associated with PTL 58, 60.

Consistent with earlier reports 61, 62, we found differential expression of inflammatory biomarkers between preterm and normal term pregnancy. Furthermore, our regression modelling illustrated that activation of mPLs is directly associated with the gestational age or labour status. Our results revealed considerable differences between gestation‐ and labour‐induced immune responsiveness. For instance, activated CD15+CD55+ granulocytes are more sensitive to labour induction, whereas CD15+CD192+ granulocytes are more likely to be affected by progression of gestation; the initiation of labour had a greater impact on the CD14+ monocyte population although gestational age contributed more to induce their CD44/CD181 activation. Because each sub‐population of blood leukocytes has a unique characteristic, we suggest that in‐depth analysis using sophisticated markers is needed to fully understand the immunological puzzle of gestational complications, that is, PTL. Recently, our group has reported an effective model to predict PTL using gene expression signatures obtained from total maternal blood leukocytes 63. To further improve the accuracy of risk evaluation for pregnancy outcomes, we propose that an integrated screening strategy incorporating clinical parameters and individual cellular characteristic, at both gene and protein levels, could be applied to existing modelling system.

In summary, we have conducted a large‐scale analysis of the changes in mPL populations and activation status during pregnancy and labour. Our results suggest that the increased number of granulocytes and NK cells as well as the activation of all mPL sub‐types provide a mechanism ensuring their increased homing to uterine tissues during term parturition. This carefully designed study used sophisticated technology which allowed the unbiased phenotyping of immune cells via multiple surface markers that distinguish their activation status during active TL and spontaneous PTL without PROM. This experimental approach could be applied to examine the immune profile of mPLs in women experiencing other gestational complications.

## Conflicts of interest

The authors confirm that there are no conflicts of interest.

## Supporting information


**Figure S1.** Experimental groups to study the activation status of peripheral blood leukocytes during human gestation and labor.

**Figure S2.** Gating strategy used for flow cytometry data analysis of different leukocyte sub‐populations.

**Figure S3.** Representative plots of the activation status for different peripheral leukocyte sub‐groups.
