# Supplementary figures and images for: Immunophenotyping and activation status of maternal peripheral blood leukocytes during pregnancy and labour, both term and preterm
Source: J Cell Mol Med. 2017 Apr 21;21(10):2386–402. doi: 10.1111/jcmm.13160 (PMC5618694; doi:10.1111/jcmm.13160)

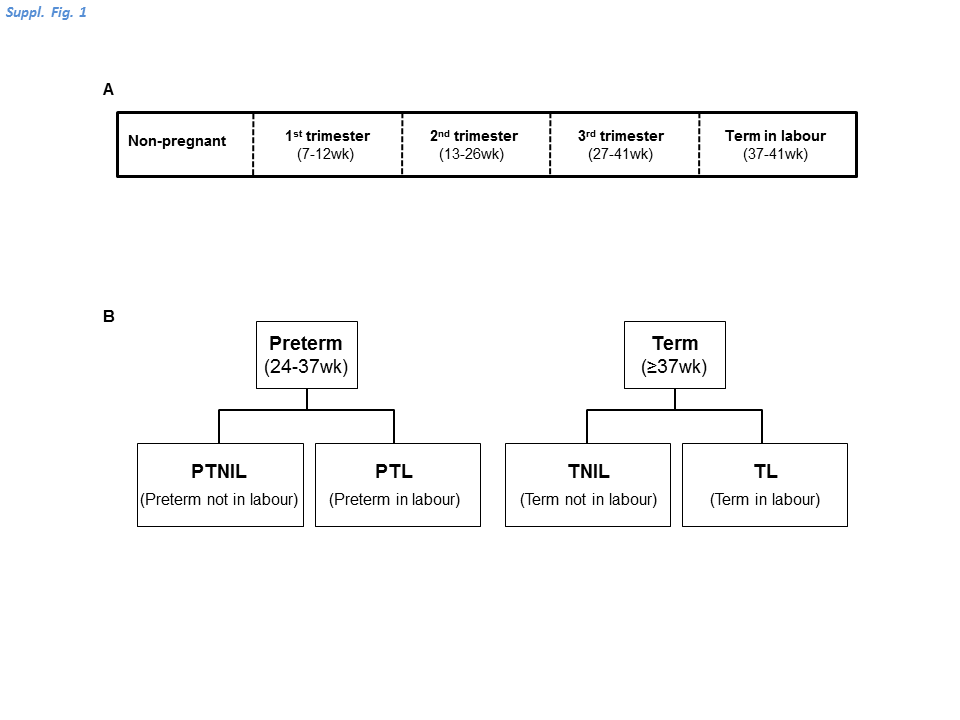

Supplement: Supplementary file 1 — Figure S1. Experimental groups to study the activation status of peripheral blood leukocytes during human gestation and labor. [file JCMM-21-2386-s001.tif]

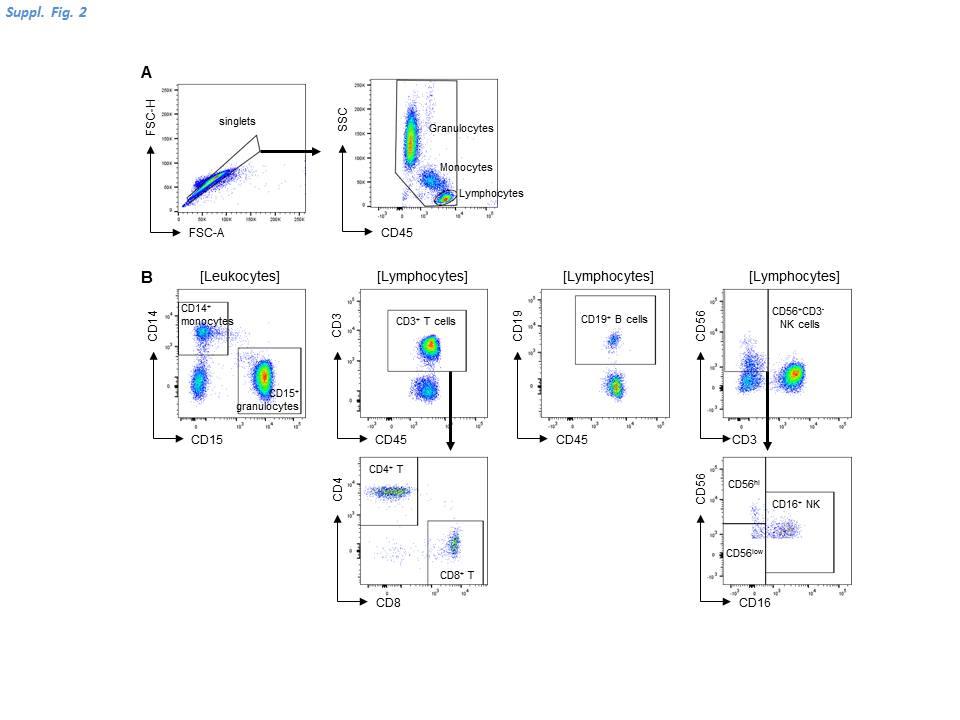

Supplement: Supplementary file 2 — Figure S2. Gating strategy used for flow cytometry data analysis of different leukocyte sub‐populations. [file JCMM-21-2386-s002.tif]

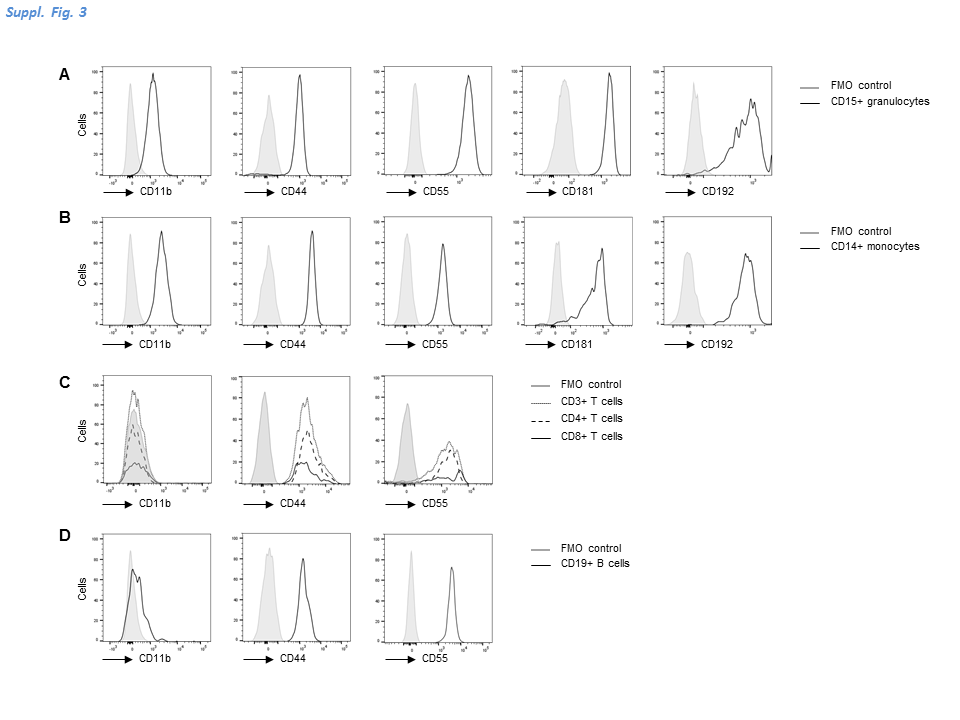

Supplement: Supplementary file 3 — Figure S3. Representative plots of the activation status for different peripheral leukocyte sub‐groups. [file JCMM-21-2386-s003.tif]
