# Supplementary material for: Immunophenotyping and activation status of maternal peripheral blood leukocytes during pregnancy and labour, both term and preterm
Source: J Cell Mol Med. 2017 Apr 21;21(10):2386–402. doi: 10.1111/jcmm.13160 (PMC5618694; doi:10.1111/jcmm.13160)
Supplement: Supplementary file 4 [file JCMM-21-2386-s004.docx]

**Supplementary Figure 1.** Experimental groups to study the activation status of peripheral blood leukocytes during human gestation and labor. A) Study 1: gestation-related changes in proportion and activation status of PLs; B) Study 2: Labor-related changes in the activation status of maternal PLs.

**Supplementary Figure 2.** Gating strategy used for flow cytometry data analysis of different leukocyte sub-populations. A) Firstly, CD45+ blood leukocytes were gated for singlets. B) Next, CD15+ granulocytes, CD14+ monocytes, CD3+ T cells, CD19+ CD3- B cells and CD56+CD3- NK cells were identified by their surface markers expression. The gates of CD4+ and CD8+ T cells were set on CD3+ T lymphocytes. Three NK subsets were recognized by their CD56/CD16 profile as CD56hi, CD56low and CD16+.

**Supplementary Figure 3.** Representative plots of the activation status for different peripheral leukocyte sub-groups. The surface expression of CD11b, CD44, CD55, CD181 and CD192 by CD15+ granulocytes (A) and CD14+ monocytes (B). The surface expression of CD11b, CD44 and CD55 on C) total CD3+ T cells (grey line), CD4+CD3+ T cells (dashed line), CD8+CD3+ T (black line); and D) CD19+ B cells. Fluorescence Minus One (FMO) control was included to identify the positively stained blood leukocytes.
